# Understanding the matrix: collagen modifications in tumors and their implications for immunotherapy

**DOI:** 10.1186/s12967-024-05199-3

**Published:** 2024-04-24

**Authors:** Rowie Borst, Linde Meyaard, M. Ines Pascoal Ramos

**Affiliations:** 1 Center for Translational Immunology, University Medical Center Utrecht, Utrecht University, Utrecht, The Netherlands; 2 Oncode Institute, Utrecht, The Netherlands; 3 Champalimaud Research, Champalimaud Centre for the Unknown, Lisbon, Portugal

## Abstract

Tumors are highly complex and heterogenous ecosystems where malignant cells interact with healthy cells and the surrounding extracellular matrix (ECM). Solid tumors contain large ECM deposits that can constitute up to 60% of the tumor mass. This supports the survival and growth of cancerous cells and plays a critical role in the response to immune therapy. There is untapped potential in targeting the ECM and cell-ECM interactions to improve existing immune therapy and explore novel therapeutic strategies. The most abundant proteins in the ECM are the collagen family. There are 28 different collagen subtypes that can undergo several post-translational modifications (PTMs), which alter both their structure and functionality. Here, we review current knowledge on tumor collagen composition and the consequences of collagen PTMs affecting receptor binding, cell migration and tumor stiffness. Furthermore, we discuss how these alterations impact tumor immune responses and how collagen could be targeted to treat cancer.

## Introduction

The extracellular matrix (ECM) is a constantly evolving structure that is produced, modified, remodeled and maintained by the cells residing within it. The ECM is dynamic and responds to changes in the local and systemic environment, making it a central player in tissue physiology and pathology such as cancer [1]. Tissues have unique ECM compositions tailored to their specific mechanical and structural demands which can be further modified in pathological conditions. The main component of the ECM are collagens consisting of 28 different types [2]. During homeostasis, the biophysical properties of collagen are critical for maintaining tissue integrity. Collagen stiffness is influenced by the size of its fibers; short fibers offer a greater range of orientation possibilities, enhancing tissue permeability, while long fibers align closely, resulting in cells organizing in the same orientation within the tissue [3]. This close alignment facilitates the formation of collagen crosslinks contributing to increased ECM stiffness [4]. In the context of tissue repair, additional collagen crosslinks serve as a protective mechanism to aid wound closure.

Cancer is viewed as a form of excessive wound-healing as similar pathways are activated in wound healing and tumorigenesis [5]. For instance, dense ECM that forms part of the scar tissue in wound healing is similar to collagen deposits known as desmoplasia in cancer, which correlate with poor prognosis [5, 6]. During tumor development, desmoplasia is induced by cancer-associated fibroblasts (CAF), macrophages and tumor cells and the produced collagen is resistant to enzymatic degradation [7–11]. CAFs are the main players in collagen remodeling in cancer. These are highly activated fibroblasts and which, in contrast to normal activated fibroblasts after tissue repair, do not undergo apoptosis or return to resting state [12]. CAFs are a heterogeneous population of cells implicated in different stages of tumor development, from primary growth to metastasis, in different tumor types [11, 12]. However, a number of studies propose an anti-tumor role for CAFs, as ablation of CAFs results in more aggressive phenotypes [13].

A hallmark of cancer is the epithelial-to-mesenchymal transition (EMT). Epithelial cells and endothelial cells secrete a laminin-rich ECM while mesenchymal cells secrete a collagen-rich ECM [14, 15]. Both EMT and desmoplasia enhance tumor stiffness by increasing the mechanical strength, density and crosslinking of collagen [16, 17]. Cells sense the mechanical properties of their surroundings and in stiffer matrices, pathways promoting proliferation, survival and invasiveness of tumor cells are triggered.

**Fig. 1:**
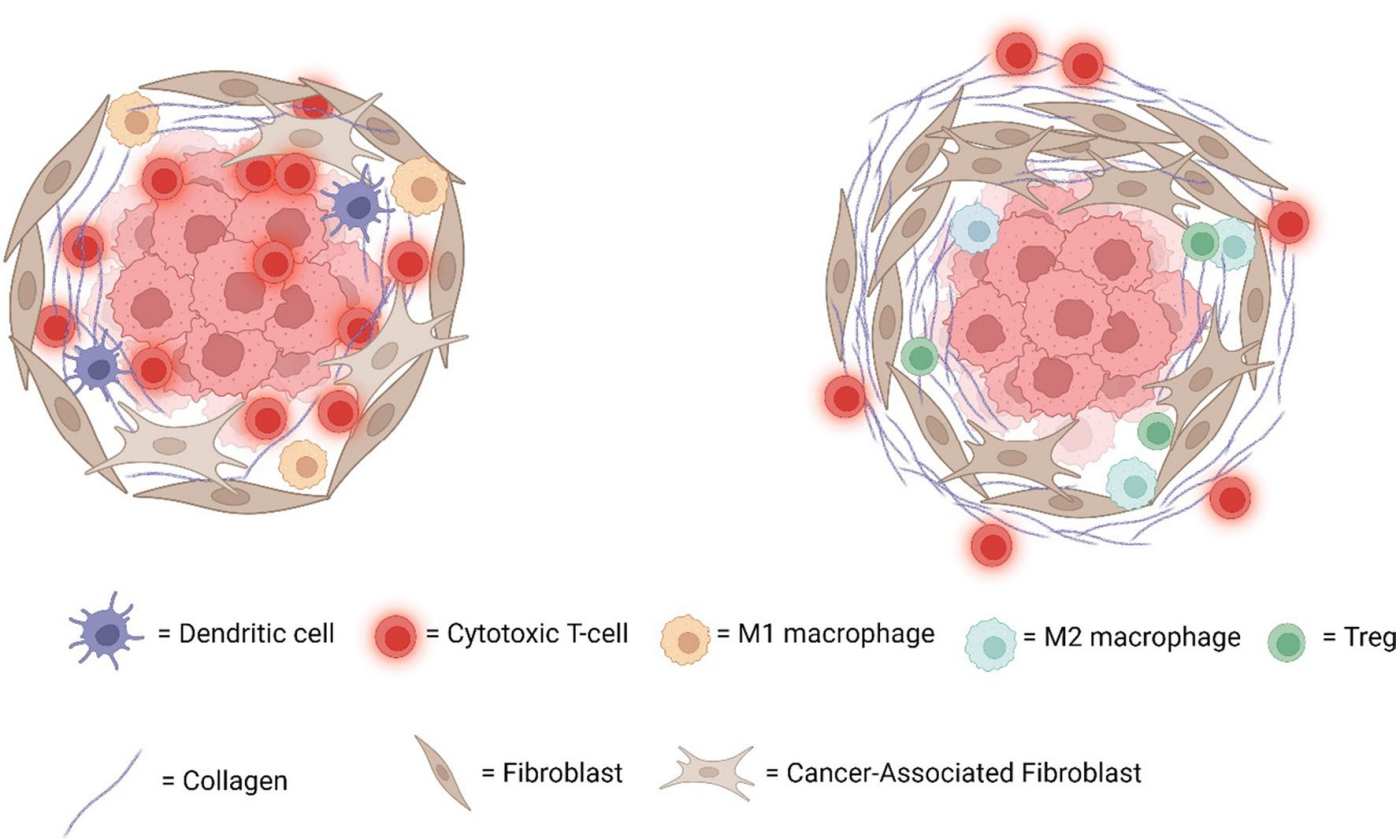
Inflamed (left) and non-inflamed tumors respond differently to immune checkpoint blockade therapy. Inflamed tumors contain more T cells, antigen presenting cells and inflammatory M1 macrophages compared to non-inflamed tumors. In non-inflamed tumors, T cells are mainly present at the tumor border and have difficulties infiltrating into the tumor. These are also characterized by more tumor-suppressing immune cells such as M2-macrophages and T-regulatory cells and high abundance of collagen produced by fibroblasts and cancer-associated fibroblasts

The ECM also influences the migration of immune cells that promote or prevent tumor growth, depending on tumor type and disease stage [7, 9, 18, 19]. T-cell migration is reduced in dense, stiff matrices compared to less dense, more flexible matrices. T cells preferentially migrate along long collagen fibers using integrin-independent migration while in disorganized collagen structures they use integrin-dependent migration [20]. Solid tumors can be classified into inflamed “hot” tumors and non-inflamed “cold” (Fig. 1). Immune inflamed tumors have a high infiltration of cytotoxic T-cells and in general are responsive to immunotherapy. Non-inflamed tumors are characterized by an absence or low numbers of infiltrated T-cells, increased collagen deposition and the presence of a stromal barrier, abnormal vasculature, lack of chemokines, hypoxia or activated oncogenic pathways [21–24]. Non-inflamed tumors have increased resistance to immunotherapy and a higher chance of disease recurrence within five years [24]. The mechanism behind the resistance is not fully understood but there is an increasing interest in the role of the ECM in immune cell infiltration of tumors. For example, the ability of T-cells to reach the tumor core of lung and ovarian tumors is hindered by the collagen alignment in the tumor periphery [25].

Collagen expression differs between different cancer types but in general tumors maintain the collagen lineament of the tissue of origin [26].However, collagens can undergo extensive post-translational modifications (PTM) [27], which can result in an almost infinite array of matrices. Manipulation of specific PTMs could provide new therapeutic possibilities to inhibit or correct localized pathological alterations to the ECM that occur in cancer or tissue fibrosis. Here, we review current knowledge on the impact of collagen and collagen PTMs on the antitumor response and their potential as therapeutic target.

### Collagen-receptor interactions affect tumor growth and anti-tumor immune responses

Cells can interact with collagens through at least six different groups of receptors, namely: (1) integrins, (2) the Discoidin Domain Receptor family (DDR1 and DDR2), (3) the mannose receptor family (4) Glycoprotein VI (GPVI) (5) Osteoclast-associated receptor (OSCAR), (6) Leukocyte Associated Immunoglobulin Like Receptor 1 (LAIR-1) [28]. The interaction of collagen with these receptors regulates diverse responses, encompassing cell adhesion, matrix metalloprotease (MMP) activity, thrombus formation, cell survival and proliferation, cytokine production, immune effector function and collagen remodeling. (Table 1).

**Table 1:** Groups of collagen receptors

Receptors	Expression	Function upon binding collagen
Integrins	All cells	Regulation of cell adhesion [29]
Discoidin domain Receptor family	Epithelial cells and mesenchymal cells	Regulation of cell migration, differentiation, proliferation, survival, ECM remodeling [30]
Mannose receptor family	Macrophages and fibroblasts	Endocytosis of collagen for lysomal degradation [31]
Glycoprotein VI	Platelets	Platelet activation [32]
Osteoclast-associated receptor	Osteoclasts, myeloid cells	Osteoclast differentiation, Monocyte survival [33]
Leukocyte Associated Immunoglobulin Like Receptor 1	Immune cells	Inhibition of immune cell activity [34]

Both immune and tumor cells bind to collagens via integrins such as α1β1, α2β1, α3β1, α4β1, α10β1, and α11β1 for migration [29, 35, 36]. Integrins expressed by tumors interact with collagen and overcomes tumor dormancy by increasing tumor cell motility. In contrast, collagen-integrin interaction on immune cells is anti-tumorigenic and promotes migration of T-cells and natural killer cells into the tumor [37]. Integrin *a3 (itga3)* mRNA expression is increased by PDAC tumor cells and negatively correlates with T-cell presence which is associated with poor prognosis [38].

The DDR family consists of receptor tyrosine kinases that bind to collagen and induce MMP secretion and regulate cellular functions [30]. DDR1 expression is positively correlated with tumor stage and promotes tumor cell proliferation, migration and invasion [39, 40]. In PDAC, the DDR1–NF-κB–p62–NRF2 cascade can be activated by cleaved collagen I which limits metabolism and growth of tumours [41]. In contrast, cleavage resistant collagen I induces proteasomal degradation of DDR1. Binding of DDR1-ectodomain to collagen mechanistically aligns collagen fibers, independent of receptor activation [42]. Tight fiber alignment can prevent immune cells from infiltrating into the tumor in breast cancer. DDR2 is overexpressed on CAFs and regulates force-mediated collagen fiber remodeling that results in a stiffer tumor microenvironment [43, 44].

The uPAR-associated protein (uPARAP/Endo180, encoded by *MRC2*) is an endocytic transmembrane receptor for collagen of the mannose receptor family [31]. uPARAP facilitates the degradation of collagen and therefore plays a crucial role in ECM homeostasis, tissue remodeling, and turnover. Macrophages and fibroblasts remodel collagens via uPARAP by targeting them to lysosomal degradation. Lower expression of uPARAP/Endo180 in metastatic melanoma and advanced urothelial cancer results in increased responsiveness to immune checkpoint blockade therapy [45].

Both GPVI and LAIR-1 recognize Glycine-Proline-Hydroxyproline motif repeats in collagens but have opposing effects on immune activation. GPVI signals through an immunoreceptor tyrosine-based activation motif (ITAM) to activate platelets resulting in thrombus formation [32]. Platelets can interact with tumor cells shielding them from shear stress in the circulation and preventing recognition by natural killer cells [46, 47]. In contrast, LAIR-1 is an immune inhibitory receptor that signals through an immunoreceptor tyrosine-based inhibition motif (ITIM) and is broadly expressed on immune cells, including T cells [34, 48]. Collagens can set a threshold for immune cell activation through LAIR-1. Collagen deposition in tumors could therefore protect tumor cells from the immune system through LAIR-1 [49].

## Changes in collagen composition during tumor progression

Collagens form a diverse family of proteins with multiple subtypes, each of which has its specific structural and functional characteristics (Table 2). The general structure of collagens consists of three polypeptide α-chains that fold into a triple helix, improving the thermal stability of the collagen. The human genome encodes for 44 different forms of α-chains to produce a total of 28 types of collagens [50]. Depending on the collagen type, this triple-helix is a homotrimer or mixture of two or three different α-chains [51]. The most common motif within α-chains is a (Gly-X-Y)n-repeat in which every third amino acid is a glycine followed by two non-glycines. The small size of glycine is crucial for the folding of the triple helix. While X–Y can be all amino acids, they most commonly are proline and hydroxyproline, respectively.

**Table 2:** Collagens organized by their subtype and tissue of origin [52]

Subtype	Collagen	Tissue of origin
Fibril-forming	I II III V XI XXIV XXVII	Bone, cartilage, skin, tendon Cartilage, vitreous body Blood vessels, bone, skin Blood vessels, bone, cornea, placenta, skin, tendon Cartilage, placenta, tendon Bone, cornea Cartilage
Network-forming	IV VIII X	Basement membrane, blood vessels, connective tissue Calcifying cartilage
Beaded-filament	VI XXVI XXVIII	Bone, cartilage, cornea, skin, Ovary, testis Basement membrane
Anchoring	VII	Basement membrane
Transmembrane	XIII XVII	Cell junctions Hemidesmosomes
FACITs	IX XII XIV XVI XIX XX XXI XXII	Cartilage, vitreous body Connective tissue Connective tissue Cartilage, papillary dermis, placenta Basement membrane, Widespread Widespread Tissue junctions

### Fibrillar collagens

The classical fibrillar or fibril-forming collagens include collagen I, II, III, V, and XI, with collagen I as the most abundant collagen throughout the body. They form long and highly organized fibrils and are the dominant component of the ECM and important contributors to cancer progression if mutated or exceedingly present [10, 53]. Long aligned fibrils provide an easy route for tumor cells to migrate out of the tumor nest while excluding immune cells [42, 54]. Pancreatic tumor cells produce unique collagen I homotrimers (a1/a1/a1) instead of the normal collagen I heterotrimers (a1/a2/a1), enhancing resistance to MMP degradation and tumor progression [38]. Homotrimeric collagen I increases proliferation of tumor cells through DDR1 and signaling through ITGA3 compared to heterotrimeric collagen I [38]. In mice models, deletion of homotrimeric collagen I or suppression of ITGA3 in tumor cells improved overall survival and tumor T-cell infiltration.

While collagen XI is a minor collagen and preferentially expressed in cartilage in homeostasis, several studies report it to be present in tumors and propose to use it as cancer-biomarker [55]. In ovarian cancer, increased expression of collagen I and XI is associated with disease progression. In non-small lung cancer, collagen XI expression in the tumor induces a negative feedback loop reducing CAF-mediated collagen remodeling and CAF migration as collagen XI sterically interferes with collagen I- integrin-binding [55].

In several cancer types, collagen V is over-expressed in non-inflamed tumors compared to inflamed tumors, in metastatic tumors compared to primary tumors and in patients resistant to cytotoxic drugs [56–60]. In contrast to most fibrillar collagens, collagen III plays a role in suppressing rather than promoting the metastatic processes such as adhesion, migration and invasion of tumor cells in a murine breast cancer model [61]. In human head and neck squamous cell carcinomas, collagen III is the most abundant collagen type in patients with dormant tumors compared to tumors from patients with additional lymph node metastases [62]. The collagen architecture of dormant tumors is characterized by wavy collagen fibers and low degree of linear organization compared to proliferative tumors [62].

### Basement membrane collagens

Network-forming collagens such as collagen IV, -VIII and -X form open network structures instead of fibers. Collagen IV is an essential part of the basement membrane and upregulated in several types of cancer promoting cell proliferation, migration, and invasion [63]. Network-collagens also play an important role in mediating platelet interaction with tumor cells and thereby enhance metastasis [47]. Collagen VIII is normally expressed in vascular smooth muscle cells (SMC) and plays an important role in vascular remodeling. High expression of collagen VIII in tumors is associated with poor prognosis, likely through SMC survival and migration, enhancing angiogenesis [64]. Lastly, expression of collagen X is high in immune-excluded triple-negative breast cancers that are resistant to anti- programmed cell death-1 (PD-1) ICB therapy [60].

### Minor collagens

Although minor collagens are less abundant in human body, they do play a crucial role in collagen structures. Beaded-filament-forming collagens such as collagen VI are closely related to basement membrane collagens. Breast cancer adipocytes upregulate collagen VI expression during tumorigenesis [65, 66]. Collagen VI is also found near vascular structures and increased in colorectal cancer [67].

Anchoring such as collagen VII and Transmembrane collagens such as XIII and XVII have a role in spatial compartmentalization and enhancing cell–cell and cell–matrix interaction, respectively [2, 68–70]. In breast cancer, collagen XIII activates the Tumor Growth Factor-β (TGF-β) pathway through B1 integrin, promoting cancer progression and metastasis [71]. In epithelial cancers, overexpression and increased ectodomain shedding of the transmembrane collagen XVII leads to tumorigenesis and is associated with poor prognosis [72]. Fibril-associated Collagens with Interrupted Triple Helices (FACIT) are important mediators in the organization of the collagen fibrils and the density of the ECM. In breast cancer FACIT collagens are highly present and inhibit fibril fusion [73, 74].

## Collagen post-translational modifications and their impact on the anti-tumor immune response

**Fig. 2:**
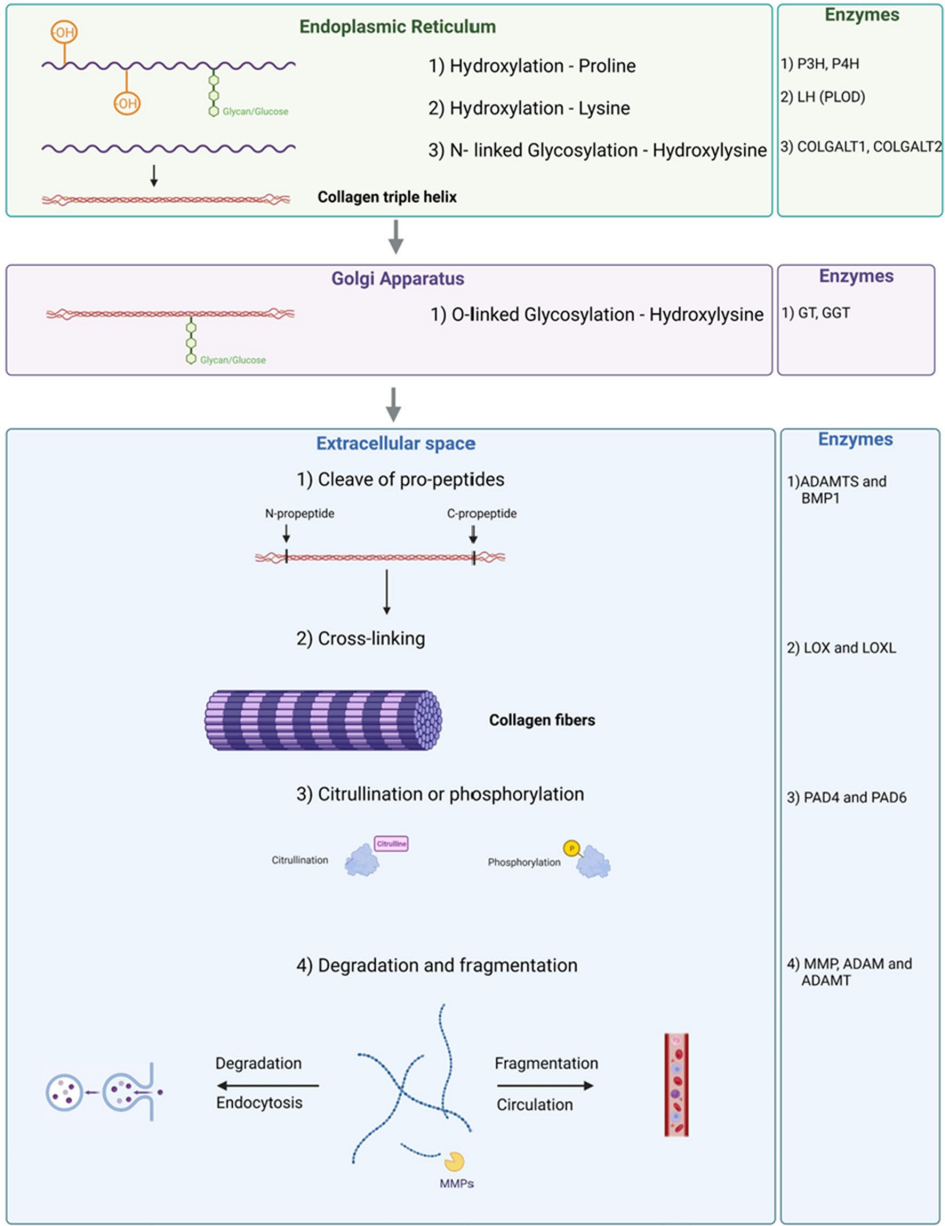
Schematic overview of collagen post-translational modifications. Hydroxylation of proline and lysine takes place in the endoplasmic reticulum while N-linked glycosylation and O-linked glycosylation of hydroxylysine take place in the endoplasmic Reticulum and golgi apparatus, respectively. The collagen triple helix is formed intracellularly and in most subtypes of collagen N- and C-propeptides are cleaved off after secretion before the collagen can be crosslinked to form collagen fibers. Collagen can also be modified by citrullination or phosphorylation. During collagen remodeling, collagen is normally degraded while in cancer also fragmentation can take place leaving collagen fragments in the circulation [2]

During collagen biosynthesis, the collagen structure undergoes several PTMs. PTMs can modify protein function by altering protein structure, protein–protein interactions, and degradation. PTMs take place intra- and extracellularly and once collagen is in its triple helical form, further PTMs such as hydroxylation and glycosylation will not occur [75, 76] (Fig. 2).

### Proline hydroxylation

The synthesis of fibrillar collagen begins with the formation of procollagen in the endoplasmic reticulum followed by proline and lysine hydroxylation and glycosylation [77]. Proline hydroxylation on fibrillar collagen by prolyl-4-hydroxylases (P4HA1, P4HA2 and P4HB) is the most frequent PTM and improves stability of the collagen triple helix by forming strong electronegative bonds. Despite the commonality of proline hydroxylation, each collagen helix exhibits a distinctive hydroxylation pattern [78]. This variability in hydroxyproline localization within the collagen structure impacts protein folding and triple helical configuration and affects protease access to the collagen structure [79–81]. Hydroxylated proline sites are important for cells to bind and interact with collagen via integrins and DDR receptors. Hence, changes in hydroxylated proline sites impact adhesion, proliferation, and cell migration [82]. Hydroxyproline and P4HA1 stabilize Hypoxia-inducible factor 1-alpha (HIF-α) enhancing the hypoxia cycle, proline synthesis and collagen deposition [83, 84] (Table 3). In The Cancer Genome Atlas (TCGA) database, high frequency of mutations in *P4HA1* are associated with lower progression free survival [85]. P4HB is overexpressed in bladder and colon cancer, increasing cell proliferation, migration and reducing apoptosis and in hepatocellular carcinoma inducing EMT [86]. For healthy collagen IV it is important to undergo PTMs such as 3-proline hydroxylation as the absence of this modification leads to platelet aggregation, which supports tumors [63].

Loss of P3H2 expression is found in breast cancer and enhances cell proliferation and is therefore pro-tumorigenic [87]. Taken together, a pattern of increased hydroxylation by prolyl-4-hydroxylases but decreased hydroxylation of 3-proline enhances tumorigenesis. This suggests that specific prolines within the collagen structure may be more susceptible to hydroxylation under pathological conditions, supporting that post-translational changes to collagen structure by specific enzymes could be used as biomarkers [82].

### Lysine hydroxylation

Besides proline, lysine also undergoes hydroxylation which stabilizes collagen triple helixes, increases the stiffness and reduces the sensitivity of collagen to proteases [88, 89]. Hydroxylation of lysine is catalyzed by lysyl hydroxylase (LH) and most commonly occurs at lysine residues in the Y-position of the Gly-X-Y sequence repeat. The α2-chains have a higher content of this repeat compared to the α1-chains resulting in more hydroxylated lysine in heterotrimeric collagens compared to homotrimeric collagens [76, 90]. Whether a lysine is hydroxylated in collagen depends on the specific amino acid sequence, activity of the hydroxylation enzyme in the collagen-producing cells and/or the collagen conformation during its exposure to the enzyme. For example, hydroxylation by LH3 is especially important in collagen IV as *LH3* deficient cell lines accumulate intracellular collagen IV, have reduced secretion and form instable triple helices [91]. Increased hydroxylation of lysine residues within telopeptides by LH2 is associated with fibrotic conditions by increasing collagen crosslinking and stiffness, protecting the collagen from degradation [89] (Table 3). This supports tumor cells by serving as a physical barrier for therapeutics and promoting metastasis [88, 89, 92]. In contrast, hypoxia decreases hydroxylation of lysine residues [78, 93]. In some tumors, mutations in human *LH2 (D689A)* lead to loss of LH activity reduction of tumor cell migration [94, 95].

### Glycosylation and glycation

Glycosylation and glycation are enzymatic and non-enzymatic reactions, respectively, of glucose, glucose metabolites and other reducing sugars with different substrates, such as proteins, lipids, and nucleic acids. Similar to other modifications, there is high variability in glycosylation patterns between different types of collagen [90]. Glycosylation is most common in less organized collagens such as collagen IV. N-linked glycosylation takes place in the endoplasmic reticulum by Collagen Beta(1-O)Galactosyltransferases. For O-glycosylation of collagens there are two glycosyltransferases that catalyze this process: hydroxylysyl galactosyltransferase (GT) and galactosylhydroxylysyl glucosyltransferase (GGT) [75]. These enzymes add glucose and galactose to the -OH group of hydroxylysine in the Golgi apparatus. The glycosylation of hydroxylysine is crucial in collagen IV and VI to assemble basement membrane. Defects in LH3 prevents intracellular tetramerization of collagen VI and its secretion [90]. Non-enzymatic glycation can also occur on fibrillar collagens, resulting in a lower number of crosslinks and reduced collagen stiffness [35]. Adding sugar molecules to collagen fibrils also impacts theirs functionality as it prohibits cell adhesion by blocking integrin-binding places on the structure [35]. Although tumor cells carry many mutations, documented mutations of genes encoding glycosyltransferases are relatively rare in tumor cells highlighting their importance for collagen stability. Overall, further mechanistic investigation is required to understand the role of collagen glycosylation in cancer and assess its potential as a novel therapeutic target.

### Propeptide cleavage

After the procollagen is released to the extracellular space, the N- and C-propeptides of fibrillar collagens are cleaved off. Cleaving of the C-propeptides of collagen I by bone morphogenetic protein 1 (BMP1) impacts the fibril formation and thus the orientation of the collagen structures. Mutations in *BMP1* are documented in individuals with gastroschisis and osteogenesis imperfecta and might potentially function as a therapeutic biomarker for individuals with cancer [96–99]. However, in case of collagen IV only the N-propeptides are cleaved off. The C-propeptides in the molecules bind head-to-head to form a network, with covalent intra- and intermolecular cross-linking into the subtype structure [77, 100]. The cleaved off N-propeptide is a non-collagenous fragment that is also known as arresten which acts as angiogenesis inhibitor [101, 102]. Arresten inhibits endothelial cell proliferation, migration and tube formation and reduces subcutaneous tumor growth in mice and suppresses squamous cell carcinoma invasion [101–103].

### Crosslinking affects matrix stiffness

Important enzymes in the regulation of fibril collagen are lysyl oxidase (LOXs) and lysyl oxidase-like (LOXL) that catalyze oxidation of lysine and hydroxylysine in a copper-dependent way [100, 104, 105]. LOX can only catalyze lysine after removal of the C-propeptides which prevents collagen from becoming anionic. High concentrations of copper ions in tumors promote LOX secretion [104]. The hypoxic TME increases tumor cell expression of LOX and promotes collagen covalent crosslinking, which increases matrix stiffness [20, 106–109] (Table 3). LOXL2 suppression in lung tumors mice, increases cytotoxic T-cell infiltration and decreases cytotoxic T-cell exhaustion [22].

Mutations in LOX are associated with colon tumor pathogenesis [110]. Despite the wealth of information available on the overexpression of LOXL2 in tumors, there are scarce data regarding the presence of genetic mutations in *LOXL2*. Mutations in *LOXL2* are identified in skin cutaneous melanoma and uterine corpus endometrial carcinoma. However, *LOXL2* mutational burden does not impact the fitness of human tumors, although it is possible that specific mutations could be important in specific types of tumors [111].

### Citrullination and receptor binding

Citrullination changes an arginine residue into a citrulline residue, which is a none-standard amino acid [112]. Intracellular protein arginine deiminases (PADs) catalyse this process and typically become active when calcium levels exceed the normal physiological concentration, for instance during apoptosis [112]. Transitioning arginines to citrullines, the reduces the positive charge of the collagen molecule, increasing hydrophobicity. PAD4 citrullinates collagen and is increasingly expressed in several types of cancer, particularly in metastases [112, 113]. PAD4 is mainly produced by neutrophils and deletion of this enzyme in mouse tumor models, results in lower neutrophil infiltration in tumors and reduced tumor progression [114]. In Rheumatoid arthritis citrullinated collagen can bind LAIR-1 as decoy ligand impairing the immunosuppressive function of LAIR-1 on T cells [115]. In cancer, impaired LAIR-1 mediated inhibition could lead to inflammation in inappropriate sites, depending on the citrullinated collagen location within the tumor. Additionally, citrullinated collagen decreases integrin-mediated cell adhesion, potentially reducing the capacity of immune cells to migrate into the tumor [116] (Table 3). A genome-wide SNP study showed a significant correlation between cutaneous-basal cell carcinoma risk and mutations in the *PAD4/PAD6* locus at 1p36 [117].

### Phosphorylation

Network-forming collagens, short-chains collagens and FACIT collagens such as collagen III, IV, V, VI, XVII XXVII can be phosphorylated [118]. Phosphorylation of collagen XVII by ecto-CK2 blocks its ectodomain shedding by Tumor necrosis factor alpha (TNF alpha)-converting enzyme (TACE), affecting the adhesion and motility of epithelial cells [119]. In squamous cell carcinoma, shed collagen XVII is suggested to promote tumor progression and invasion [72]. Therefore, it is tempting to speculate that collagen phosphorylation has a protective role in epithelial cancers but further research has to be conducted to elucidate this.

### MMP degradation and collagen fragments

The ECM undergoes constant remodeling involving collagen cleavage by proteases such as matrix metalloproteases (MMPs), a disintegrin and metalloproteinases (ADAMs) and ADAM with thrombospondin motifs (ADAMTs) [120, 121]. These enzymes directly influence the biological characteristics and functions of collagen by uncovering cryptic sites, releasing collagen-bound growth factors and degrading collagen [122]. Compared to intact collagen fragmented collagens are unstable and therefore more prone to degradation. However, collagen fragments still have a bioactive role by binding to cell surface receptors regulating numerous biological processes in physiological and pathological situations [123, 124]. MMP-1, 8 and 13 also known as collagenase 1, 2 and 3 have a pro-tumorigenic role by cleaving fibrillar collagens and enhancing tumor cell motility. MMP-2 and MMP-9 cleavage activates latent TGF- β and produces collagen fragments which in turn induces TGF-β secretion. TGF-β has an inhibitory effect on cell proliferation in early stages of cancer and is also a key factor in fibrosis [125, 126]. Collagen I fragments cleaved by MMP1, 2, and 14 activate the DDR-1 receptor enhancing tumor growth in pancreatic cancer, thereby reducing patient survival [41]. Collagen I fragments cleaved by MMP-1 and MMP-9 have an inhibitory effect on T-cell receptor activation and IFN-y secretion through LAIR-1 signaling [127].

Large-scale genomic studies have delved into the potential genetic alterations of MMPs across a spectrum of human malignant tumors from diverse origins. These studies have specifically revealed MMP8 as a frequently mutated gene in human melanoma [128]. Functional analysis of the identified mutations verified that all mutations result in loss-of-function of MMP8, contributing to melanoma progression. These findings conclusively establish *MMP8* as a tumor-suppressor gene. Additionally, parallel studies have expanded these observations to other MMP-related metalloproteinases, such as *ADAMTS15* that is genetically inactivated in human colorectal cancer [129].

**Table 3:** Amino acids in collagen with their corresponding post-translation modification, added group and enzyme involved. The outcome of overexpression of each modification is described in the last column

Amino acids	PTM	Group	Enzymes	Outcome if increased
Proline	Hydroxylation (ER)	–OH	P4Hs, P3Hs	Hypoxia
Lysine	Hydroxylation (ER)	–OH	LH1, LH2, LH3	Increased glycosylation, crosslinking and fibrosis
Hydroxylysine	N-Linked (ER) O-linked (GA) Glycosylation	Glucose or galactose	COLGALT1, COLGALT2, GT, GGT	Increased crosslinking, matrix stiffness
Lysine/Hydroxylysine	Oxidation (ES)	–O	LOX, LOXL1, LOXL2, LOXL3, LOXL4	Fibrosis, matrix stiffness
Arginine	Citrullination (ES)	Citrulline H_2_O to NH4 +	PAD4	Role in protein folding, apoptosis, TGF-β Pathway, receptor binding

## Collagen post-translational modifications as potential novel therapeutical targets in cancer

Numerous potential treatments, including antibodies and small molecule inhibitors, are currently studied for their ability to target enzymes and PTMs involved in ECM remodeling in tumors (Table 4). Targeting intracellular PTMs could inhibit collagen secretion and deposition, reduce stiffness and change the collagen architecture, thereby improving immune cell migration and penetration into the tumor mass. Various rate-limiting steps in collagen deposition were explored, including the targeting of proline hydroxylases. Knocking down P4HA1, P4HA2, and HIF-α reduces collagen deposition in primary breast cancer tumors, consequently preventing metastases [130]. Additionally, small molecules targeting P4HA1 reduce tumor growth in colorectal cancer models possibly through inhibition of MMP1 [131]. Aspirin targets P4HA2 by decreasing its gene transcription which results in reduced collagen deposition and tumor growth in hepatocellular carcinoma [132].

Collagen fibers in tumors are characterized as linear and compact due to the high level of deposition and post-translational crosslinking. This physical restructuring of collagen progressively stiffens the ECM leading to extensive biochemical and biomechanical changes, affecting cell signaling and tumor tissue three-dimensional architecture [133]. Therefore, targeting collagen crosslinking might be a good anticancer therapeutic strategy. In mice, anti-BMP1.3 treatment reduces expression of collagen I, LOX and TGF-β leading to a reduced overall scar size and improved cardiac function in a model of cardiac fibrosis [134]. This therapy shows significant potential in preventing fibrosis with minimal adverse effects. Investigating its potential effects on already established fibrotic tumors or in preventing metastases would be of interest. LOX/LOXL inhibitors, specifically LOXL2 inhibitors, are used in cancer and fibrosis to prevent collagen crosslinking [135]. In mice and clinical studies, LOXL targeting results in low toxicity and adverse effects, but yielded limited clinical benefits [135]. In preclinical cancer models, inhibiting LOXL2 does result in a reduction in metastasis but not in reduced primary tumor size [136]. In the clinic, LOXL2 inhibitors are used before surgical intervention to reduce metastasis [137]. Given that only the crystal structure of LOXL2 is solved, the potential of inhibitors targeting other LOXL enzymes has yet to be explored. Inhibitors of LOX enzymatic activity such as beta-aminopropionitrile (BAPN) were tested in combination with PD-1 treatment in mouse models leading to tumor reduction and increased T-cell infiltration [138], however the clinical use of BAPN is impeded by concerns regarding toxicities [139]. Another approach to reduce LOX/LOXL activity is to target copper which is an important cofactor for LOXL functionality [140]. Inhibiting copper results in anti-angiogenic, anti-fibrotic activities, however, the mechanism of LOXL-regulation by copper is poorly understood [141]. In preclinical mouse models, treatment with a copper chelator reduces the levels of myeloid-derived suppressor cells and increases CD4 + T-cell infiltration in tumors [140].

Extensive experimental and clinical data associate MMPs with tumor invasion, neo angiogenesis, and metastasis, positioning MMPs as promising pharmacologic targets for cancer therapy [122]. Numerous MMP inhibitors demonstrated promise as anti-cancer treatments in pre-clinical studies [142]. Unfortunately, none of them progressed significantly in clinical trials due to severe adverse effects, including musculoskeletal pain and inflammation [143]. MMP inhibitors that lacked specificity did not succeed in clinical trials, but current efforts are focused on developing more specific antibodies and inhibitors [144].

Of note, most inhibitors are still clinically tested in metastatic cancer while it is hypothesized that MMP inhibition would be more effective in early stages of tumor progression [144]. Since then, the understanding regarding the diversity of MMPs, the intricacy of their mechanisms, and the cross-reactivity of certain inhibitors with the ADAM and ADAMTS families has increased. Endogenous MMP inhibitors such as Thrombospondin-1 (TSP-1) regulate MMP-2 and MMP-9 activity reducing tumor growth in pre-clinical tumor models [145]. However, the function of TSP-1 in angiogenesis and tumor progression remains disputable in certain cancers and may be organ specific [146]. While TSP-1 is identified as an inhibitor of both processes, while in others, it is characterized as a stimulator [145, 147–151]. Additionally, MMPs can modulate the immune system by regulating chemokines and altering their activity [152]. MMP9 plays a pivotal role in promoting tumorigenesis across various cancer types. Inhibiting MMP9 leads to enhanced chemotaxis through elevated expression of CXCL10, coupled with increased T-cell activation triggered by higher levels of IL12p70 and IL-18 expression [153]. In preclinical models, the combined administration of anti-MMP9 and anti-PDL1 results in increased intra-tumoral T-cell diversity characterized by larger CD4/CD8 memory and effector cell populations, along with an enhanced Th1 responses [153].

The increasing body of evidence for PADs in cancer progression [154] has resulted in a growing interest towards targeting PADs and citrullination as potential therapeutic targets. Tumor cells can produce PAD4 and high PAD4 expression is found in patients’ blood and malignant tumor tissue [155]. In mice, PAD4 deletion in combination with ICB therapy results in increased presence and activation of CD8^+^ T cells, reduced tumor growth and lung metastasis compared to ICB treatment only [114]. Whether this effect is due to PAD4-mediated collagen citrullination has not yet been investigated in tumors.

Another approach to improve cancer treatments based on tumoral ECM characteristics, is using fusion proteins with a collagen binding domain (CBD) carrying bioactive-inhibiting cues, immune chemoattracts or radioactive substances. For example, recombinant protein containing the EGFR binding fragment of cetuximab improved by a CBD resulted in specific targeting to and penetration into squamous carcinoma A431 cell xenografts [156]. A similar approach was used with CBDs fused to immune checkpoint inhibitor antibodies and to IL-2 [157]. Both CBD-fused IL2 and CBD-conjugated checkpoint inhibitors showed enhanced antitumor efficacy and reduced associated toxicity compared with their unmodified counterparts in several tumor models. In addition, CBD fusion to IL-12 is described as result in systemic toxicity reduction and synergy with immune checkpoint inhibitor therapy [158]. This targeting strategy could also leverage collagen PTMs making this approach more tumor specific. Specific ECM components and PTMs are highly expressed in areas of active tumor invasion and thus could be used as targets. This strategy has the potential to augment the efficacy of radiation, chemotherapy, or targeted therapy by concentrating drugs, or antitumor biologics specifically at active tumor sites, thereby reducing their dispersion in healthy tissues [159].

**Table 4:** inhibitors and drugs targeting collagen modifications

Therapy target	Name drug	Drug format	Disease	Research stage
P4HA1	Diethyl-pythiDC	Small molecule inhibitor	Colorectal cancer	Preclinical [131]
P4HA2	Aspirin	Small molecule inhibitor	Hepatocellular carcinoma	Preclinical [132]
P4H	EDHB	Small molecule inhibitor	Breast cancer	Preclinical [160]
BMP1.3	Anti-BMP1.3	mAb	Myocardial infarction	Preclinical [134]
LOX	BAPN (β-aminopropionitrile) PXS-5505	Irreversible inhibitor Small molecule inhibitor	Cancer Pancreatic cancer	Preclinical [139] Preclinical [161]
LOXL2	Simtuzumab GS341	mAb pAb	Fibrosis, Cancer	Phase II [162] Preclinical [135, 163]
LOXL2	PXS-S1A PXS-S2A PAT-1251 PXS-5382A Epigallocatechin gallate (EGCG)	Small molecule inhibitors	Fibrosis, heart failure, glaucoma, oncological and angiogenic diseases	Phase II [135] Phase II [135] Phase I [164, 165] Phase I [135] Phase I [166]
Copper	tetrathiomolybdate (TM)I d-penicillamine (D-pen)	Copper chelators	Cancer Breast cancer	Phase I/II [140, 141, 167] Phase II [136, 168]
Dual LOX/LOXL	PXS-5153A CCT365623	Inhibitor	Fibrosis Cancer	Preclinical [169] Preclinical [170, 171]
MMP9 MMP9 MMP14 MMP1, MMP2, MMP3	Andecaliximab (GS-5745) AB0041, AB0046, DX-2400 Single Chain Fragment Variables	mAb mAb mAb mAb	Cancer, Colorectal cancer Breast, melanoma, sarcoma Breast cancer	Phase III [172, 173] Preclinical [144, 174] Preclinical [175] Preclinical [176]
PAD4 PAD4 PAD4 PAD4	TDFA TDCA Cl-amidine F-amidine GSK199, GSK484 JBI-589	Selective Irreversible small molecule inhibitors Reversible inhibitors Small molecule	Inflammatory disorders and Cancer Cancer	Preclinical [177] Preclinical [178, 179] Preclinical [177] Preclinical [114]

## Conclusion and future perspectives

Immune therapy revolutionized cancer treatment options. However, not every tumor responds well to this treatment, especially tumors with high desmoplasia and low immune cell infiltration are resistant to therapy. Collagen deposition in tumors acts as a physical barrier to therapeutic treatment. This barrier is not only passive, keeping immune cells out, but can also actively protect the tumor cells specially when altered by certain PTMs. To enhance cancer treatment for non-responders, immune therapy could be combined with therapies targeting the ECM of tumors.

To implement ECM targets in future treatment of cancer patients, more studies should focus on when the ECM changes from being tumor suppressive to tumor promoting and which PTMs play an important role in this process. Promoting increased immune cell infiltration through the breakdown of the ECM may also create an opportunity for tumor cells to disseminate throughout the body. Hence, the course of treatment and the tumor stage should be meticulously assessed and determined. Characterizing different types of collagens, PTMs and the abundance of PTM associated enzymes could aid in stratifying patients who may benefit from ICB alone or in combination with ECM targeted therapies. Targeting collagens and collagen-modifying enzymes for oncological purposes is intricate, given the widespread presence of collagen throughout the body. However, understanding the spatial heterogeneity and temporal dynamics of collagen PTMs in different types of solid tumors has the potential to refine the selective targeting of tumor stroma and bolster anti-tumor immune responses.

## Data Availability

Not applicable.

## Abbreviations

ECM: Extracellular matrix
ICB: Immune checkpoint blockade
PTM: Post-translational modification
CAF: Cancer-associated Fibroblasts
EMT: Epithelial-to-mesenchymal transition
DDR: Discoidin domain receptor
GPVI: Glycoprotein VI
OSCAR: Osteoclast-associated receptor
LAIR-1: Leukocyte Associated Immunoglobulin Like Receptor 1
MMP: Matrix metalloproteases
ITAM: Immunoreceptor tyrosine-based activating motif
ITIM: Immunoreceptor tyrosine-based inhibiting motif
uPARAP: UPAR-associated protein
SMC: Smooth muscle cells
PD-1: Programmed cell death-1
TGF-β: Transforming growth factor beta
FACIT: Fibril-associated Collagens with Interrupted Triple Helices
P4H: Prolyl-4-hydroxylases
P3H: Prolyl-3-hydroxylases
HIF-α: Hypoxia-inducible factor 1-alpha
TCGA: The cancer genome atlas
LH: Lysyl hydroxylase
GT: Galactosyltransferase
GGT: Galactosylhydroxylysyl glucosyltransferase
BMP-1: Bone morphogenic
LOX: Lysyl oxidase
LOXL: Lysyl oxidase-like
PAD: Protein arginine deiminases
TNF-α: Tumor necrosis factor alpha
TACE: Tumor necrosis factor alpha (TNF alpha)-converting enzyme
ADAM: A disintegrin and metalloproteinases
ADAMT: A disintegrin and metalloproteinases with thrombospondin motifs
BAPN: Beta-aminopropionitrile
TSP-1: Thrombospondin-1
CBD: Collagen binding domain
mAb: Monoclonal antibody
